# A longitudinal case series of IM ketamine for patients with severe and enduring eating disorders and comorbid treatment‐resistant depression

**DOI:** 10.1002/ccr3.3869

**Published:** 2021-04-04

**Authors:** Terry Schwartz, Mary Ellen Trunko, David Feifel, Emily Lopez, Danika Peterson, Guido K. W. Frank, Walter Kaye

**Affiliations:** ^1^ Department of Psychiatry University of California San Diego San Diego CA USA

**Keywords:** depression, eating disorders, ketamine, treatment

## Abstract

Severe and enduring eating disorders (EDs) have the highest mortality rate of all psychiatric illnesses (Arch Gen Psychiatry, 2011, **68**, 724), especially when comorbid with treatment‐resistant depression (TRD) (Psychiatr Res, 2016, **244**, 45). We report on four patients with enduring EDs and TRD treated with repeat ketamine over 12 + months, showing improvement in depression with only modest changes in ED symptoms.

## Graphical abstract

Severe and enduring eating disorders (EDs) have the highest mortality rate of all psychiatric illnesses, especially when comorbid with treatment‐resistant depression (TRD). We report on four patients with enduring EDs and TRD treated with repeat ketamine over 12 + months, showing improvement in depression with only modest changes in ED symptoms.

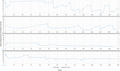

## 1 INTRODUCTION

Eating disorders (ED) have high comorbidity rates with mood and anxiety disorders.^1^, ^2^ Such comorbidity adversely impacts engagement in treatment and worsens outcomes for patients with EDs. Specifically, a lifetime diagnosis of major depressive disorder (MDD) is a negative predictor of BMI and recovery in AN.^2^

There is a growing literature, supporting the use of Ketamine for severe treatment‐resistant depression (TRD)^3^ and associated suicidality. TRD has varied definitions, but generally refers to patients with MDD who have failed several trials of monotherapy antidepressants (and often failed multiple augmentation strategies). Controlled trials of Ketamine for treatment of TRD have consistently shown rapid and effective response with IV infusion of Ketamine.^4^, ^5^ Both the robust and the rapid time frame of response (24‐48 hours) have exciting potential for severely depressed patients, at least in the short term. Additionally, case reports of repeat IV Ketamine at variable intervals (weeks to months) for maintenance have preliminary evidence of relative safety and efficacy out to 1 year.^6^

It is not known whether ketamine could be helpful for the treatment of ED symptomatology or whether reduction of depressive symptomatology could mediate improvement of EDs. To our knowledge, there has been only one previous study using ketamine to treat patients with EDs.^7^ That case series included underweight and normal‐weight individuals with AN or BN. The effect of ketamine infusion on weight was inconclusive but, in the responders (60%), depression scores improved and measures for compulsive behaviors improved. Therefore, the effectiveness of ketamine in EDs has not been sufficiently studied. We selected patients with EDs who had a very long‐standing TRD as well as severe and enduring ED illness.^8^ Our aim was to determine whether ketamine would improve depression in EDs and, also improve ED symptom severity.

## 2 METHODS

We selected four female patients who had been chronically ill with an eating disorder for more than 7 years duration. All patients had completed an eating disorder PHP in the previous 6 months, and were having ongoing significant eating disorder and mood symptoms. All patients were in standard outpatient treatment for the bulk of the data collected, with the exception being case 4, who was completing the final few weeks of 3 day IOP program after first ketamine treatment. In addition, subjects met current criterial for treatment‐resistant depression (TRD) without psychotic features. Each had previously failed more than four trials of antidepressant medications of adequate dose and duration. Subjects met DSM‐5 criteria for Major Depressive Episode and an Eating Disorder^9^ by clinical interview by 2 senior psychiatrists and moderate or severe level of depression was confirmed by scores on the Beck Depression Inventory‐I ( BDI)^10^ being greater than 20. Suicidality was assessed clinically. Additionally, a response of 2 or 3 on question 9 (suicidality question) on BDI was flagged on BDI for immediate follow‐up check in with psychiatrist.

Subjects completed a baseline self‐report assessment and were weighted before the injection of ketamine. These assessments include the BDI, Spielberger state‐trait anxiety inventory (STAI),^11^ and Eating Disorder examination questionnaire (EDE‐Q).^12^ Depression, anxiety, and ED symptoms were also longitudinally assessed by BDI, STAI, and EDE‐Q preinjection, 24 hours postinjection, 3‐ and 7‐days postinjection. This schedule of measures began after treatment 2 for case 2, and any other missing data points were related to patient not completing measures as requested. Self‐rating scales were used for clinician and patient convenience. Time between follow‐up injections was based (primarily) on individual patient's time frame in which they started to relapse in depression symptoms. Patients were also asked for narrative descriptions of their symptoms and function throughout the treatment course.

As per previously published guidelines,^4^ patients deemed appropriate for a trial of ketamine treatment were provided with relevant information about the treatment, including the fact that the treatment was not currently FDA approved and not reimbursed by insurance. Patients were provided with a written informed consent document for treatment which described potential risks and limitations in detail as well as reasonable expectations of ketamine treatment for depression. Patients were given an opportunity to ask questions which were addressed by the prescribing physicians.

An experienced Ketamine physician (DF) and RN were in the room, and monitoring patient mental status and vitals signs, per UC San Diego Hospital protocol. Ketamine was administered mainly IM, with the first 2‐3 doses administered IV for cases 3 and 4. IM Ketamine has similar data to support equivalent dosing, efficacy, and safety as with IV Ketamine.^13^ IM was used given the convenience of IM over IV dosing. IV dosing requires op room and presence of an anesthesiologist. IM, given slower rates of absorption, can be administered by an MD, without an anesthesiologist present. Of note, in cases 3 and 4, the first several treatments were IV and done with an anesthesiologist present.

The standard dosing of 0.5 mg/kg was the initial dose. Subsequent doses were titrated to response, side effects, and safety.^14^ Some of our cases had an additional injection of 0.3‐0.4 mg/kg to alternate deltoid 24 hours after the first dose, if previous dose of 0.5 mg/kg was well tolerated, but, only partially effective, or ineffective. Patients remained on all their previous medications. Ketamine dose and frequency were determined by clinical status, magnitude, and duration of previous dose response, and safety/side effects. Safety was maintained by monitoring vital signs, side effects, cognitive/psychiatric status, and physical symptoms for approximately 2‐3 hours after injection. The main side effect was the classic ketamine dissociative “trip “that lasted 30‐90 minutes, post‐treatment sleepiness, and occasional mild headache. The patients were then sent home with a preplanned relative or friend driver. There were no vital sign abnormalities.

## 3 RESULTS

### 3.1 **Case 1**: **history**

**FIGURE 1:**
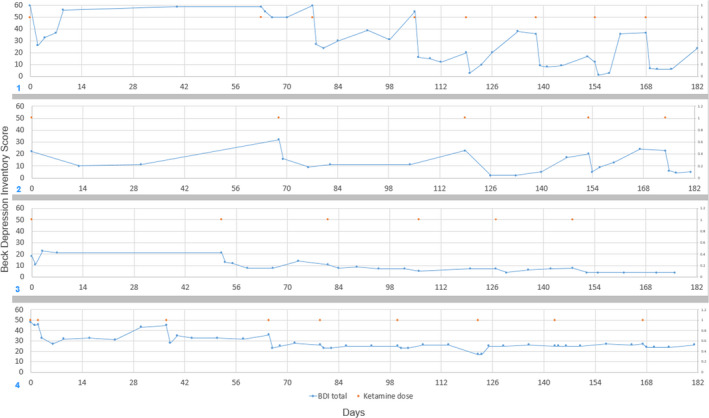
Beck depression inventory‐I vs days from initial dose. Cases 1‐4, respectively. Orange dots = ketamine administration day. Blue dots BDI measurement (y‐axis) and day (x‐axis). Days with both Blue and Orange dots depict the preadministration BDI

A 49‐year‐old single white female with chronic restricting type AN (AN‐R), TRD, and post‐traumatic stress disorder (PTSD). She had been severely depressed for the past 10 years Figure 1, Table 1. She had failed 9 adequate antidepressant trials, including combination therapies with several augmenting agents. She also failed a full course of rTMS (Transcranial Magnetic Stimulation). She had chronic suicidality and was socially isolated. In addition, she failed multiple eating disorder programs including residential, partial hospital, and outpatient levels of care. Her BMI at the time of initial Ketamine treatment was 19. Her medications during Ketamine treatment were as follows: aripiprazole 10 mg and fluoxetine 40 mg. She was in weekly therapy, seeing a dietician monthly, and a psychiatrist monthly for the duration of the trial.

**TABLE 1:** Summary of BMI, and qualitative changes in STAI‐state and EDE‐Q eatcon/restraint results

Case	Age	ED	Other diagnosis'	BMI initial	BMI final	STAI‐state	EDE‐Q control/restraint
1	49	AN‐R	Bipolar II‐depressed, PTSD	19	22.6	Improved, partial sustained	Varied, slight decrease
2	30	AN‐R	MDD	17.5	22.2	Improved, sustained	Improved, sustained
3	33	EDNOS‐BP	MDD	25.2	25.8	Improved, partial sustained	Improved, partial sustained
4	35	EDNOS‐BP	MDD, BLPD/O	37.8	39.2	Limited improvement, not sustained	Limited improved, not sustained

#### 3.1.1 Ketamine dose

The IM injection dosing was adjusted to 0.4 mg/kg right deltoid and 0.4 mg/kg to the left deltoid 24 hours apart, per protocol, this was the minimal dosing to provide adequate benefit, with minimal side effects.

#### 3.1.2 Depression and anxiety

There were improvements in her mood, energy, and general well‐being within 24 hours. (see Figure 1, panel 1). She continued to get the injections every 3‐5 weeks over the next 18 months. Her BDI was initially 46. She had a rapid decrease in her BDI to the low 20s, increasing to a maximum in the mid‐twenties as time between injections increased, she continued to show a BDI response throughout her course, with no apparent tolerance or reduction in response. Her STAI‐state anxiety symptoms were reduced (from low 70s pretreatment, to low 60s range postinjections). The STAI‐trait anxiety scale remained in low to mid 70s. Her suicidality was significantly clinically reduced from “wanting to be dead” for many years, to only having occasional passive suicidal thoughts.

#### 3.1.3 ED symptoms

Eating disorder examination questionnaire weight and shape concern also showed improvement postinjection, with partial rebound to previous level before the next treatment. This correlated clinically with her reports that her food flexibility and obsessionality were reduced, allowing her to be more flexible in her eating. Her BMI increased over the course of treatment from 19 to 22.6.

#### 3.1.4 Impressions

Clinically, there was a substantial reduction in hopelessness and suicidal thoughts. She remained stable during a period of a major life stressor/loss for her. Some ED symptoms showed a mild improvement. Her weight increased, and her food variety improved. She also had improved energy, motivation, and drive, allowing her to return to the workforce, and is no longer on disability.

### 3.2 Case 2: history

A 30‐year‐old female with a chronic course of AN‐R and TRD for over 12 years, with two previous serious suicide attempts culminating in her admission to inpatient psychiatric unit in August of 2014. At the time of her hospitalization, her BMI was 17.5. She was overwhelmed by simultaneously trying to cope with her ED and TRD. She has had 10 + trials of antidepressants, combined with various augmentation strategies, with limited to no response. She was taking escitalopram 20 mg, was in weekly outpatient therapy, and saw psychiatrist every 4‐6 weeks during the trial.

#### 3.2.1 Ketamine dose

Initial dosing was 0.5 mg/kg IM. This was gradually increased at subsequent treatments to two IM ketamine injection 0.5 and 0.4 mg/kg 15 minutes apart (left and right deltoid, respectively) to allow for increased magnitude and duration of mood response and minimizing side effects.

#### 3.2.2 Depression and anxiety

Her BDI preketamine was 43. (Figure 1, panel 2). After 2 IM shots of 0.3 mg/kg and 0.5 mg/kg IM, 24 hours apart, her BDI was reduced to 17. Her subsequent BDIs showed clinically meaningful reduction after each injection, with a lowering of “maximum BDI” between treatments. Over time, her BDIs were frequently below 10. Her response did not diminish over time. After ketamine was stopped, she maintained stability of mood. She did not have any relapse of her ED since starting the Ketamine, nor did her suicidality return. Her ED and anxiety symptoms showed some improvements, mostly coinciding with postinjection times. Clinically, she reported a dramatic improvement in her mood, energy, and hopefulness. She was no longer suicidal, and in fact, was hopeful about her future. Additionally, the patient noted that she experienced a significant reduction in anxiety associated with eating certain foods, including desserts. Her follow‐up outpatient Ketamine IM injections were 0.5, and 0.3 −0.4 mg/kg, to the right and left deltoid, respectively, 15‐20 minutes apart.

#### 3.2.3 ED symptoms

She has a notable reduction in the EDE‐Q “eating concerns” subscale (2.5‐1). Here, EDE‐Q restraint subscale showed a less robust improvement, though clinically her weight stabilized. Her weight remained stable at 98% of IBW, and, she began to have regular menstrual periods for the first time in 12 years. Her BMI increased from 17.5 when she was first hospitalized to 22.2 over the next year. During this time, she was also treated with Lexapro 10 mg a day.

#### 3.2.4 Impressions

While her weight and shape concerns remained unchanged related to ketamine treatments, her “eating concerns**”** improved, as above. This is consistent with her reports of being more able to eat appropriately, despite the ED thoughts, with less prolonged and intense guilt afterward. Finally, her STAI‐state values also appear to follow her mood improvement. Her STAI‐Trait values remained unchanged.

### 3.3 **Case 3:** history

A 33‐year‐old female with Other specified feeding and eating disorders (OSFED)^9^ and Major depressive disorder, recurrent as well as having affective dysregulation starting in her teenage years. She had failed 3 prior antidepressant trials. Prior to a trial of ketamine, she has presented to a treatment program with a BMI of 23.2, with AN‐like symptoms including restriction and very rigid, limited food choices. She engaged in compulsive exercise, for 3‐4 hours most mornings, and had no history of binge eating or purging by vomiting. During 4 months of PHP/IOP for her ED, the patient's depression improved significantly but she made minimal progress with ED behaviors and lost insurance coverage for the program. Following discharge, the patient experienced significant weight fluctuations. She began purging by vomiting. During the subsequent 9 months, she gained 30 lbs. (BMI = 27.8), while continuing the excessive exercise, vomiting several times daily, and restricting. The patient denied bingeing and she had begun using alcohol and frequent THC. She continued treatment with sertraline 200 mg and aripiprazole 10 mg, but depression returned. The patient was then referred for ketamine treatment. Her BMI at the time of initial Ketamine treatment was her pre‐Ketamine BMI was 25.2. She was in outpatient care during the trial, seeing a psychiatrist intermittently, and declined individual therapy.

#### 3.3.1 Dose

Ketamine 0.5 mg/kg IM to deltoid throughout her treatment.

#### 3.3.2 Depression and anxiety

Patient had consistent reduction in BDI and STAI‐state post‐Ketamine injections. While these measures of depression and anxiety increased between doses, there was a reduction of peak (between doses) BDI and STAI‐state values.

#### 3.3.3 ED symptoms

She also had consistent reduction in all 4 EDE‐Q subscales after treatments.

#### 3.3.4 Impressions

Toward her final treatments, she lost her job and had a relationship break up. Her BDI, scores increased, but, responded well to ketamine. Her narratives during these tougher times reported her ability to stay strong and positive, which surprised her. Clinically, this was correlated with reduced intensity, frequency severity, and duration of eating disorder behaviors. Her BMI was 25.2 pre‐Ketamine, and 25.8 at the end of treatment. What is notable about this, is that her weight stabilized during the 12 months she was getting ketamine.

### 3.4 **Case 4**: **history**

A 35‐year‐old female with widely fluctuating weight history including both AN‐R and obesity, chronic and unremitting MDD, PTSD, somatization, and Borderline personality disorder (BLPD). Patient developed AN and reports a lifetime low BMI = 13.5 in her 20s. She partially weight restored to a BMI of 17.8. She maintained that weight for 4 years, until she was treated for a back injury with steroids for 2 years, which resulted in a serious weight gain, with eventual high BMI of 47.5. Subsequently, she began severely restricting intake, and lost 60 lbs over a couple months, to a BMI = 34.5. After a month‐long fast, she was hospitalized for medical complications, spent 1 month in residential treatment, and then entered an ED partial hospital program (PHP), with step down to intensive outpatient program (IOP). In addition to frequent fasting and excessive exercise she had used diet pills but had never purged by vomiting. Patient had been in and out of PHP/IOP treatment several times and continued to have wide weight fluctuations, with limited change in mood, despite multiple adequate antidepressant trials and augmentation strategies. Even during a 9 + month period with normalized eating patterns and BMI stabilization at 25.5, the patient continued to complain of significant residual depression and PTSD symptoms. Patient was taking venlafaxine XR 150 mg, lamotrigine 200 mg, and topiramate (the latter for migraine headaches). Her mood an anxiety symptoms were unchanged throughout ED treatment, and multiple med trials. She was in her final 3 weeks of a 3 day IOP program at the time of first injection, and otherwise had weekly therapy and every 2‐3 months psychiatry follow‐up per her HMO.

#### 3.4.1 Dose

Patient had initial dose of ketamine IV, 0.5 mg/Kg. She was then converted to IM. Her dose was progressively increased, since lower doses were only partially effective and only lasted <2 weeks. Her max and maintained dose was 0.85 mg/kg weekly injections. She tolerated this dose very well, with mild sedation postinjection.

#### 3.4.2 Depression and anxiety

Both her mood (BDI) as well as her state anxiety STAI showed clear and repeated response to Ketamine treatment, including times of BDI dropping to under 5, with her pretreatment BDI at 60. She also showed periods of time where her BDI remained lower than her pretreatment baseline. Her responses were more variable after 6 months, and less sustained. During this time, she reported an increase in stress, PTSD symptoms. Her suicidal thoughts varied throughout the treatment period and were more related to interpersonal stressors.

#### 3.4.3 ED symptoms

All of her EDE subscales showed little to no response to ketamine treatments.

#### 3.4.4 Impressions

While this person had initial benefit to her mood and anxiety, the magnitude and consistency of response were limited. This patient was very sensitive to interpersonal stressors, and such events would often limit or eliminate any potential Ketamine benefit. There was no benefit to her eating disorder symptoms.

## 4 DISCUSSION

Ketamine reduced depression in all four cases we reviewed with TRD and severe and enduring EDs—as measured by BDI and clinical assessment. During the trial, none of the patients were hospitalized or admitted to a higher level of care. There were no suicide attempts by any patient for the trial duration. The magnitude of the response did not diminish over time with repeat dosing. BDI response was partially sustained between treatments for Patient 1 and Patient 2 over the 12 + month course. Patients 1 and Patient 2 had significant changes in their course of illness, as measure by functionality, BDI, and clinical assessment. Of note, both patients had been stagnant in their depression and eating disorder treatment.

In this small sample of patients with severe and enduring EDs complicated by TRD, Ketamine IM (dosing 0.5‐0.80 mg/kg) administered, with repeat dosing at 4‐6 weeks intervals resulted in clinically meaningful changes in depression and to a lesser degree anxiety and eating disorder symptoms. All patients moved at least 1 level down in the classification system used for BDI scores and level of depression (10).

The improvement in depression was most notable and sustained in Cases 1 and 2 (more classic AN‐R), both of whom had more robust and sustained clinical improvements in depression, anxiety, and 2 clinically relevant EDE‐Q subscales (EDE‐Q eatcon and restraint). Furthermore, both case 1 and case 2 have had remarkable improvements in their previously downward functioning and quality of life trajectories. The longitudinal course of their depression and ED varied, however, for case 2, the patient is in full remission from her 12‐year battle with both AN‐R and TRD. These changes are sustained at 3 years since initiation of Ketamine treatment and 1.5 years out from her last Ketamine dose. For case 1, the patient has continued intranasal Ketamine, continues to work, and is off disability. Patient (case 1) has maintained a BDI in the mild‐moderate range, and, while she is not in full remission from her ED her weight has been in her goal weight range consistently for the past year, which had not been achieved in the past.

Treatment‐resistant depression comorbid with chronic restrictive eating disorders are very difficult patients to treat in terms of response of either disorder. In these few cases, we saw improvement in both disorders, particularly for the 2 classic AN‐R patients. These results, while very preliminary, offer some hope for patients with chronic AN‐R and TRD. Given the combined high morbidity and mortality rate of combined AN‐R and TRD, having a rapid acting effective treatment for 1 or both disorders could help improve outcomes in this patient type, who otherwise have a very poor prognosis.

In summary, this pilot study found that IM ketamine appeared to efficacious for TRD in this group of patients with severe and enduring EDs. However, the effects of ketamine on ED symptoms were more modest. The 2 patients (cases 1, 2) with AN‐R had the most robust and sustained responses in their ED and TRD. Both these patients also had clinically significant reduction and elimination of suicidality. It remains unknown whether a reduction in mood symptoms contributed to an improvement in ED symptoms, or there was a specific effect on core AN symptoms.

## 5 LIMITATIONS

This was an open‐label, naturalistic case series, without a placebo control.

## CONFLICT OF INTEREST

The data that support the findings of this study are available from the corresponding author, [TS], upon reasonable request. The work described in this manuscript was performed as part of routine clinical care and was not funded by any third party. None of the authors have any conflict of interest. However, David Feifel MD PhD now owns a clinic that administers Ketamine for depression. He did not own this company during the treatment and data assessment for these case reports.

## AUTHOR CONTRIBUTIONS

TS: involved in psychiatrist providing care 2/4 patients, primary author. MET: involved in psychiatrist working with 2/4 patients. DF: involved in Ketamine Provider and data collection. EL: provided help with graphics. DP: involved in data set management. WK: assisted with data analysis and writing. GKWF: data review and manuscript preparation.
